# Dysfunctional Intestinal Microvascular Endothelial Cells: Insights and Therapeutic Implications in Gastrointestinal Inflammation

**DOI:** 10.1097/IN9.0000000000000043

**Published:** 2024-05-29

**Authors:** Ji Seok Park, Gail A. M. Cresci

**Affiliations:** 1) Department of Gastroenterology, Hepatology and Nutrition, Digestive Disease Institute, Cleveland Clinic, Cleveland, Ohio, USA; 2) Department of Inflammation & Immunity, Lerner Research Institute, Cleveland Clinic, Cleveland, Ohio, USA

**Keywords:** intestinal microvascular endothelial cells, leukocyte homing, inflammatory bowel disease, MAdCAM-1, α_4_β_7_ integrin, vedolizumab

## Abstract

The intestinal microvascular endothelium plays a crucial role in orchestrating host responses to inflammation within the gastrointestinal tract. This review delves into the unique aspects of intestinal microvascular endothelial cells, distinct from those of larger vessels, in mediating leukocyte recruitment, maintaining barrier integrity, and regulating angiogenesis during inflammation. Specifically, their role in the pathogenesis of inflammatory bowel diseases, where dysregulated endothelial functions contribute to the disease progression is reviewed. Furthermore, this review discusses the isolation technique for these cells and commonly used adhesion molecules for in vitro and in vivo experiments. In addition, we reviewed the development and therapeutic implications of a biologic agent targeting the interaction between α_4_β_7_ integrin on T-lymphocytes and MAdCAM-1 on gut endothelium. Notably, vedolizumab, a humanized monoclonal antibody against α_4_β_7_ integrin, has shown promising outcomes in inflammatory bowel diseases and other gastrointestinal inflammatory conditions, including chronic pouchitis, immune checkpoint inhibitor-induced colitis, and acute cellular rejection post-intestinal transplantation.

## 1. Introduction

The vascular endothelium, arising from the mesoderm, constitutes the innermost layer of the entire vascular system, including both blood and lymphatic vessels ^[1]^. Microvascular endothelial cells play a critical role in host responses to inflammation by mediating leukocyte homing, transmigration to the interstitial space, and angiogenesis ^[1,2]^. These cells function as “gatekeepers” by secreting pro-inflammatory cytokines and expressing selective adhesion molecules in the vascular bed to recruit leukocytes into inflammatory foci ^[3]^. These features are different from endothelial cells lining large blood vessels such as artery and vein ^[4]^. Compared to endothelial cells residing in large vessels, microvascular endothelial cells are generally thought to be more responsive to proinflammatory stimuli and growth factors ^[5]^.

These unique roles of the microvascular endothelial cells in inflammation have been investigated in various gastrointestinal diseases with inflammation, such as inflammatory bowel disease (IBD) ^[6,7]^. The intestinal endothelium serves as a critical barrier between the bloodstream and tissue microenvironment. Microvascular endothelial cells in the context of IBD exhibit significant barrier disruption and altered levels of adhesion molecules ^[7]^. Increased permeability of endothelial junctions leads to the leakage of inflammatory mediators and leukocytes, contributing to the transmigration of leukocytes from the bloodstream into the mucosa. Increased expression of adhesion molecules such as intercellular adhesion molecule-1 (ICAM-1), vascular cell adhesion molecule-1 (VCAM-1), and mucosal addressin cellular adhesion molecule-1 (MAdCAM-1) facilitates leukocytes binding to endothelial cells, leading to their infiltration into the inflamed tissue microenvironment in intestine ^[7]^. Leukocytes can also degrade endothelial junctions through protease upregulation and secretion ^[7]^. Furthermore, in IBD a decreased expression of endothelial nitric oxide synthase (eNOS) compromises vasodilation and increases oxidative stress due to nitric oxide dysregulation ^[8–12]^. Moreover, Toll-like receptors (TLRs) expressed on endothelial cells represent another layer of complexity in this inflammatory condition. TLRs respond to bacterial antigens in the gut, mediating endothelial-dependent inflammation and host-commensal interactions ^[13]^. Dysregulation of endothelial TLR-signaling in IBD can trigger disease-related inflammation ^[14]^. Microvascular endothelial cells respond rapidly to stimulation with the TLR agonists, lipopolysaccharide (LPS) from *Escherichia coli (E. coli)* via TLR4 and flagellin from *Salmonella* spp. via TLR5, by expressing ICAM-1, VCAM-1 and cytokines such as IL-6 and IL-8 ^[15]^. Lastly, microvascular endothelial cells in IBD exhibit dysregulated angiogenesis which is important in tissue repair and healing ^[7]^. These cells respond to inflammatory cytokines and growth factors, especially vascular endothelial growth factor-A (VEGF-A), to proliferate and migrate to form new blood vessels. The abnormal angiogenesis during inflammation leads to blood vessels that are leaky, immature, and hyperthrombotic, thereby exacerbating tissue injury and promoting recruitment of inflammatory cells ^[16,17]^.

Overall, these multilayered dysfunctions to endothelial cells play a crucial role in the pathogenesis of IBD, contributing to inflammation and disease progression. However, the time-consuming and challenging process of isolating and culturing primary microvascular endothelial cells^[15]^ has posed a barrier to conducting robust in vitro investigations using these cells, compared to the relative ease of working with epithelial cells or mesenchymal cells.

## 2. Methods of investigation

Microvascular endothelial cells can be investigated using both in vitro and in vivo experiments ^[18,19]^. In vitro culture with primary microvascular endothelial cells is ideal for testing direct effects of various agents on endothelial cell integrity and/or activation. Isolation of primary human intestinal microvascular endothelial cells (HIMEC) is a tedious process involving several steps over the course of weeks to finally achieve confluent monolayers. Typically surgically resected intestine or colon undergoes a series of processing including enzymatic and mechanical isolation techniques ^[4,6,18]^. Platelet/endothelial cell adhesion molecule-1 (PECAM-1), also known as CD31, is used to identify and sort microvascular endothelial cells from mesenchymal cells which are then cultured using sterile culturing protocols.

Several molecular markers expressed by microvascular endothelial cells, ICAM-1, VCAM-1, E-selectin, PECAM-1, and MAdCAM-1 can be quantified or visualized in both in vitro and in vivo experiments ^[1,6,20–22]^. For example, PECAM-1 is a 130-kDa transmembrane glycoprotein which is present on the surface of platelets, monocytes, macrophages, neutrophils and endothelial cells intercellular junction ^[23]^. This adhesion molecule mediates adhesion between endothelial cells, and plays a major role during angiogenesis. PECAM-1 also regulates the adhesion cascade between endothelial cells and inflammatory cells, and therefore can be included as one of the readouts from in vitro or in vivo experiments.

MAdCAM-1 is preferentially expressed on endothelial cells in intestinal mucosa, submucosa, and Peyer’s patches and plays a critical role in leukocyte homing to the inflammation foci ^[24,25]^. MAdCAM-1 regulates both rolling and adhesion of lymphocytes to intestinal endothelial cells through binding of α_4_β_7_ integrin or l-selectin expressed on lymphocytes ^[26]^. This interaction is important for lymphocyte homing to the lamina propria which plays a critical role in intestinal inflammation. In both Crohn’s disease (CD) and ulcerative colitis (UC), intestinal endothelial expression of MAdCAM-1 is upregulated ^[27,28]^. MAdCAM-1 messenger RNA (mRNA) and protein expression are inducible with TNF-α, IL-1β, or LPS activation in vitro ^[29]^. MAdCAM-1 also exhibits unique expression features associated with intercellular interaction as opposed to ICAM-1 and E-selectin. Its expression is dependent on culture duration and cellular density, underscoring the important role of intercellular interaction among endothelial cells in the expression of MAdCAM-1 ^[29]^.

Previously, microvascular endothelial cells and adhesion molecule expressions were investigated in both mice and primary human intestinal microvascular endothelial cells following exposure to ethanol ^[19]^. Mice subjected to acute ethanol exposure (5gm/kg) exhibited increased MAdCAM-1 expression and decreased PECAM-1 expression in the small intestine compared to those exposed to placebo. Furthermore, intestinal microvascular endothelial cells isolated from a patient without any intestinal inflammatory processes and from a patient with UC were cultured and monolayers were exposed to ethanol and LPS for 1 hour. Interestingly these exposures induced the expression of MAdCAM-1 and ICAM-1 only in the HIMEC from UC patient; and interestingly, butyrate co-treatment during ethanol and LPS exposure rescued the induction of these adhesion proteins by. These findings suggest that HIMEC with inflammatory memory can be induced by ethanol and LPS and underscore the feasibility of the involvement of gut microbial factors in regulating their activation.

## 3. Targeting MAdCAM-1 and α_4_β_7_ integrin for the treatment of IBD and other inflammatory gastrointestinal diseases

Previous studies have demonstrated that monoclonal antibodies against α_4_β_7_ integrin or MAdCAM-1 inhibit migration of lymphocytes into the lamina propria and Peyer’s patches which blunts intestinal inflammation in murine models ^[30,31]^. These investigations eventually led to the development of novel anti-integrin biologic agents for the treatment of IBD ^[7,32,33]^. Two biologic agents, vedolizumab and natalizumab, are approved by the United States Food and Drug Administration (FDA) for the treatment of CD ^[33]^. Vedolizumab is a humanized monoclonal antibody against α_4_β_7_ integrin approved for both CD and UC. Natalizumab is a humanized monoclonal antibody against α_4_ integrin, which is now infrequently used for CD due to the risk of developing progressive multifocal leukoencephalopathy, a rare but fatal neurologic disease. Vedolizumab does not cross into cerebrospinal fluid and has not been associated with the development of progressive multifocal leukoencephalopathy. Vedolizumab can be used for both induction and remission of both CD and UC. It is a treatment option for patients who do not respond to, lose response to, or have contraindications to anti-TNF biologics agent by offering a different mechanistic target ^[33]^.

The efficacy of vedolizumab for the treatment of CD and UC was studied in the GEMINI 1 and 2 clinical trials ^[34,35]^. The GEMINI 1 trial was a two-integrated randomized, double-blind, placebo-controlled trial of vedolizumab in patients with active UC. In the induction therapy trial, 274 patients (cohort 1) received vedolizumab or placebo at weeks 0 and 2, and 521 patients (cohort 2) received open-label vedolizumab at weeks 0 and 2, with the disease evaluation at week 6. In the maintenance therapy trial, patients who had a response to vedolizumab at week 6 from either cohort were randomly assigned to vedolizumab (every 8 or 4 weeks) or placebo for up to 52 weeks. The response rates at week 6 were 47.1% and 25.5% among patients in vedolizumab group and placebo group, respectively (*P* < 0.001). Patients who continued to receive vedolizumab had higher clinical remission rates (41.8% in the 8 weeks group, 44.8% in the 4 weeks group) than patients who switched to placebo (15.9%) (*p* < 0.001). Similarly, the GEMINI 2 trial was also a two-integrated randomized, double-blind, placebo-controlled trial in patients with active CD. In the induction trial phase, 368 patients (cohort 1) received vedolizumab or placebo at weeks 0 and 2, and 747 patients (cohort 2) received open-label vedolizumab at weeks 0 and 2, with the disease evaluation at week 6. In the maintenance therapy trial, patients who had a response to vedolizumab at week 6 from either cohort were randomized to receive vedolizumab (every 8 or 4 weeks) or placebo for up to 52 weeks. The clinical response rates at week 6 were 14.5% and 6.8% for patients in vedolizumab group and placebo group, respectively (*P* = 0.02). Patients who continued vedolizumab had higher clinical remission rates (39.0% in the 8 weeks group, 36.4% in the 4 weeks group) than patients who switched to placebo (21.6%) (*P* < 0.001 and *P* = 0.04 for the two interval groups, respectively). Based on these two clinical trial results, FDA approved vedolizumab to treat CD and UC in 2014 ^[36]^.

The key advantage of this gut-specific anti-integrin monoclonal antibody is that its side effect profile does not include serious systemic infection which is associated with conventional systemic immunosuppression from corticosteroids or anti-TNF biologic agents ^[37]^. Inflammation within the intestinal tissue leads to the upregulation of MAdCAM-1 expression on the gut endothelial cell surface to interact with α_4_β_7_ integrin on the T lymphocytes. As the monoclonal antibody binds to α_4_β_7_ integrin, the molecule cannot interact with MAdCAM-1, effectively inhibiting the rolling and adhesion of gut-homing T-lymphocytes across the intestinal microvascular endothelium. This specific inhibition downregulates inflammation in both CD and UC, and in potentially other gastrointestinal diseases with inflammatory process that involves intestinal T lymphocytes.

Chronic pouchitis is one of the gastrointestinal diseases with chronic inflammation that may benefit from vedolizumab targeting. Restorative protocolectomy with ileal pouch-anal anastomosis (IPAA) is a common procedure in patients with severe UC that requires colectomy ^[38]^. Approximately half of these patients develop idiopathic inflammation within 5 years after IPAA ^[39]^, leading to increased stool frequency, abdominal pain, and impaired quality of life ^[40,41]^. One of the characteristic findings of an inflamed pouch is lymphocyte infiltration ^[42,43]^, suggesting that vedolizumab may be effective in the treatment of chronic pouchitis. The efficacy of vedolizumab in chronic pouchitis was investigated in a phase 4, double-blind, randomized trial by the EARNEST study group ^[38]^. Patients were assigned to receive vedolizumab or placebo on day 1 and at weeks 2, 6, 14, 22, and 30. In the intention-to-treat analysis, which included 102 patients who underwent randomization, the incidence of remission at week 14 was 31% with vedolizumab and 10 % with placebo (*P* = 0.01), which was the primary end point of the trial. The incidence of remission remained to be higher at week 34 in the vedolizumab group (35%) compared to the placebo group (18%). These results suggest that vedolizumab may be considered as a treatment option in chronic pouchitis by inhibiting T-lymphocyte migration across the microvascular endothelial cells ^[44]^.

Immune checkpoint inhibitor (ICI)-induced colitis is another inflammatory condition that can be mitigated by vedolizumab. ICIs, specifically cytotoxic T-lymphocyte activator-4 (CTLA-4), programmed cell death protein 1 (PD-1), and programmed cell death ligand 1 (PD-L1) inhibitors have changed the survival outcomes and therapeutic guidelines in multiple malignancies, including but not limited to, small cell lung carcinoma, renal cell carcinoma, and melanoma ^[45–48]^. However, ICIs can cause side effects. ICIs lead to a widespread T-lymphocyte activation that is not tumor-specific ^[49]^. When this widespread activation is combined with the depleted regulatory T-lymphocyte from ICI, it can cause immune-related adverse events (irAEs) in multiple organ systems ^[50]^. One of the commonly involved organs is the gastrointestinal tract, manifesting as transient diarrhea in mild cases or severe enterocolitis in life-threatening cases. Corticosteroids are used as a first line treatment in ICI-induced colitis ^[51]^, but there have been investigations on vedolizumab use in severe cases. In a retrospective, multi-center case series, 28 patients were included who had ICI-induced colitis, refractory to corticosteroids and/or infliximab (anti-TNFα agent), and subsequently treated with vedolizumab ^[50]^. In patients who only failed corticosteroids, vedolizumab resulted in a successful clinical response rate of 95%. In patients who failed both corticosteroids and infliximab, vedolizumab led to a successful clinical response rate of 67%. Based on these results, vedolizumab can be considered as a therapeutic option in severe, steroid-refractory, ICI-induced colitis ^[51]^.

Acute cellular rejection (ACR) after intestinal transplantation is another inflammatory process in which vedolizumab may be considered. Intestinal transplantation is the least performed of currently performed organ transplants ^[52]^, and has the lowest graft survival rates compared to other solid organ transplants ^[53,54]^. One main cause of graft loss is ACR, characterized by gut homing of recipient T-lymphocyte after priming with antigens from the donor ^[55–58]^. Migration of the activated recipient T-lymphocytes from the endothelium to the lamina propria result in epithelial cell damage and trigger ACR. In a murine model of intestinal transplant, the allografts were infiltrated with many T-lymphocytes with α_4_β_7_ integrin, and administering a blocking antibody specific for β_7_ integrin reduced the cellular infiltrate and subsequently inhibited ACR ^[59]^. The use of vedolizumab in ACR after intestinal transplant in human has been limited to case reports and case series, but it can be considered as a therapeutic option given its gut-specific mechanism, specifically targeting α_4_β_7_ integrin ^[57,58,60]^.

## 4. Summary and future studies

Microvascular endothelial cells serve a pivotal and distinct function in the context of gut inflammation, primarily by facilitating leukocyte recruitments to sites of inflammation through the regulation of the adhesion molecule expressions. The dysfunction of these microvascular endothelial cells, characterized by dysregulated adhesion molecule expressions, impaired nitric oxide synthesis, altered TLR signaling, and uncontrolled angiogenesis are essential components in the pathogenesis of IBD. These cells can be studied by quantifying or visualizing key adhesion molecules, particularly MAdCAM-1. Despite the inherent challenges associated with investigating intestinal microvascular endothelial cells, previous investigations have led to the development of biologic agents by targeting the interaction between α_4_β_7_ integrin on T-lymphocytes and MAdCAM-1 on the gut microvascular endothelium. The FDA-approved drug vedolizumab has shown favorable outcomes for other diseases, such as chronic pouchitis, ICI-induced colitis, and ACR after intestinal transplantation. Future studies are needed to prove the efficacy of vedolizumab in more rigorously designed, randomized, controlled clinical trials to expand its indications by the FDA approval. Such investigations will be essential to elucidate the full spectrum of vedolizumab’s therapeutic potential in various inflammatory gastrointestinal diseases.
